# Obesity and early-onset colorectal cancer risk: emerging clinical evidence and biological mechanisms

**DOI:** 10.3389/fonc.2024.1366544

**Published:** 2024-05-02

**Authors:** Peng Xu, Zuo Tao, Hua Yang, Cheng Zhang

**Affiliations:** ^1^ Department of General Surgery, General Hospital of Northern Theater Command, Shenyang, Liaoning, China; ^2^ Department of Breast Surgery, The First Hospital of China Medical University, Shenyang, China; ^3^ Department of General Surgery, Xinqiao Hospital, Army Medical University, Third Military Medical University, Chongqing, China

**Keywords:** obesity, EOCRC, metabolic syndrome, intestinal microbe, inflammation

## Abstract

Early-onset colorectal cancer (EOCRC) is defined as diagnosed at younger than 50 years of age and indicates a health burden globally. Patients with EOCRC have distinct risk factors, clinical characteristics, and molecular pathogenesis compared with older patients with CRC. Further investigations have identified different roles of obesity between EOCRC and late-onset colorectal cancer (LOCRC). Most studies have focused on the clinical characteristics of obesity in EOCRC, therefore, the mechanism involved in the association between obesity and EOCRC remains inconclusive. This review further states that obesity affects the carcinogenesis of EOCRC as well as its development and progression, which may lead to obesity-related metabolic syndrome, intestinal dysbacteriosis, and intestinal inflammation.

## 1 Introduction

Obesity is characterized by an excessive accumulation of adipose tissue and defined in terms of body mass index (BMI) (1). An imbalance between energy intake and energy consumption was correlated with the gain in weight. Obesity is defined as a BMI value > 30 kg/m^2^ through the formula weight (kg)/height^2^ (m^2^) (30.0-34.9, grade I; 35.0-39.9, grade II; and ≥40, grade III) (2). In the last decades, the prevalence of obesity has been increasing and obesity has become an epidemic problem (3). Obesity is also strongly correlated with environment and socio-economic status, which may accelerate the development of cancer through gene-environment interaction (3). In China, obesity rates tripled between 1991 and 2006 (4), with 10.5% prevalence of obesity in men in China (5).Obesity is a well-known risk factor for colorectal cancer (CRC). The risk of CRC increased by 1.2-1.5 folds in patients who were overweight and by 1.5-1.8 folds in patients with obesity (6). Previous investigations have implicated that obesity acted differently between early-onset colorectal cancer (EOCRC) and late-onset colorectal cancer (LOCRC) (7, 8). EOCRC is defined as CRC diagnosed at younger than 50 years of age (9). Moreover, CRC occurring in patients older than 50 years is defined as LOCRC (10). EOCRC accounts for nearly 10% of newly diagnosed cases of CRC; an increase in global incidence of EOCRC has been noted globally, especially in high-income regions (11). Adhari Al Zaabi et al. explained obesity as a modifiable risk factor for EOCRC (12). Obesity resulted in an increasing incident rate of EOCRC (13, 14). However, the clinical and biological plausible explanation of the association of obesity with EOCRC needs further research (15).

In this review, we first provide an overview of differences between EOCRC and LOCRC, association between obesity and EOCRC, and summarizing the main clinical differences and biological mechanisms, with an emphasis on: 1) clinical evidence between obesity and EOCRC 2) obesity-associated metabolic disorder in EOCRC, 3) obesity-induced dysfunction of intestinal microbiota, and 4) obesity-induced inflammation. It is crucial to elucidate the association between obesity and EOCRC for the development of preventive, diagnostic, and therapeutic strategies against cancer.

## 2 Difference between early-onset colorectal cancer and late-onset colorectal cancer

### 2.1 Different onset processes of early-onset colorectal cancer

**Figure 1:**
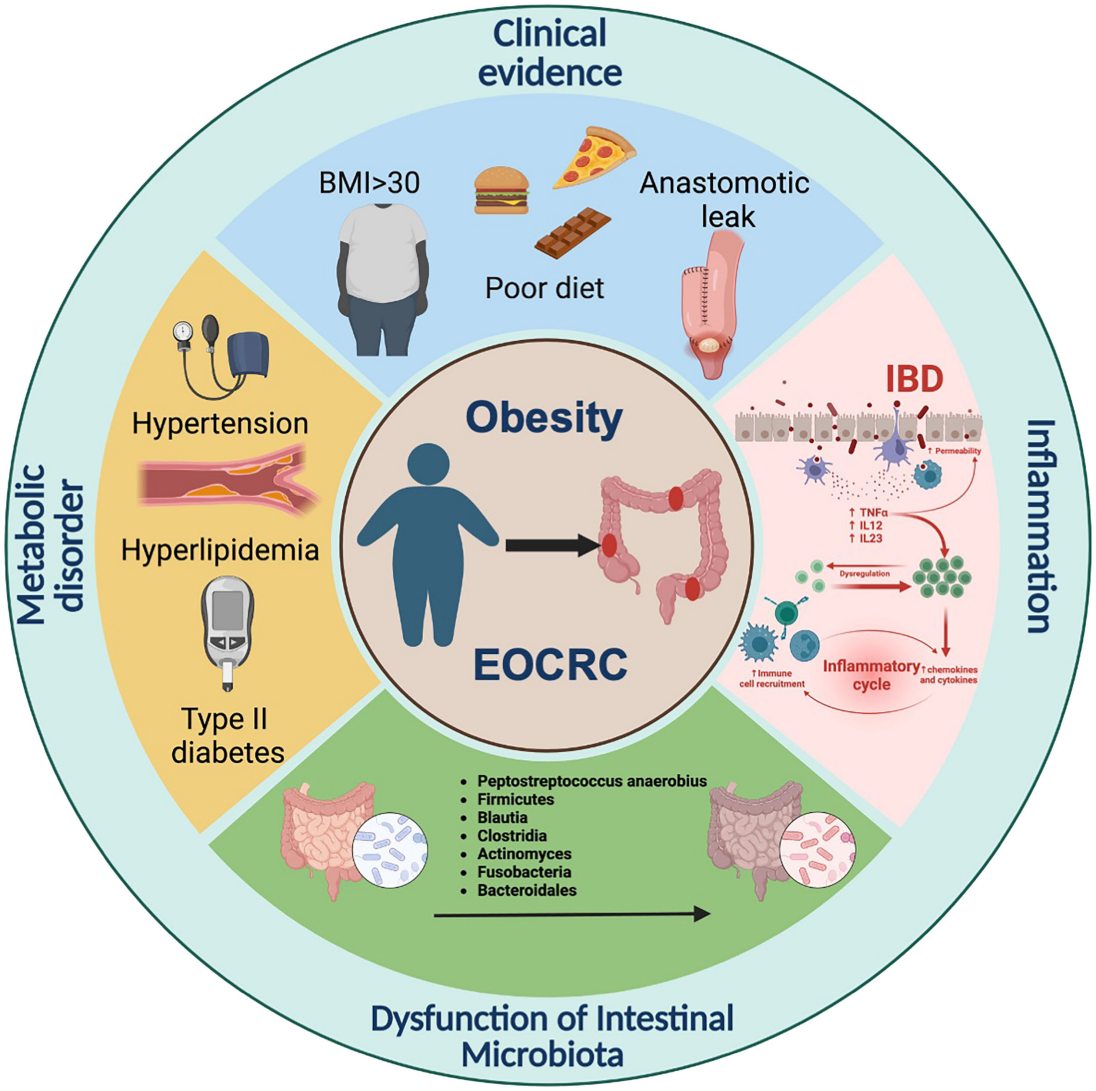
Differences between EOCRC and LOCRC on clinical features.

CRC results from the accumulation of multiple genetic changes, however, most CRCs share only a few mutations (16). The main differential features between CRC of both age groups have been summarized (**Figure 1**). Genetic features in EOCRC are distinct from LOCRC. The incidence of EOCRC may be attributed to the accumulation of multiple rare genetic variants. Chromosome instability (CIN), microsatellite instability (MSI), and CpG island methylation (CIMP) are the three main processes involved in the onset and development of CRC (17). CIN is one of the most common forms of genomic instability, and is associated with poor survival, metastases, and resistance to cancer therapy (18). Along with higher rates of advanced histologic features and later stages, CIN is observed in most patients with EOCRC (19). CIN is also associated with the progression of adenoma to carcinoma, while MSI and CIMP are associated with the progression of sessile serrated polyps to invasive carcinomas (16). MSI results from mismatch repair deficiency and is associated with the primary loss of function of mismatch repair proteins (MLH1, MSH2, MSH6, and PMS6) (20). Research has showed that EOCRC is enriched in MSI, which is a distinct genetic pathway for CRC carcinogenesis (21, 22). One-third of EOCRC patients with MSI have been diagnosed with a defined inherited syndrome (23). EOCRC patients with MSI are also less responsive to 5fluorouracil-based chemotherapy (24, 25). Along with hereditary characteristics of EOCRC, Lynch syndrome (LS) is regarded as the most common hereditary cancer syndrome related to MSI and occurs at a higher frequency in EOCRC (17, 21). In LS, the most frequently mutated genes were MLH1 and MLH2, while mutations in MSH6 and PMS2 were rather lower (26). Correlations between germline deletions of epithelial cell adhesion molecules and silencing of MSH2 have also been observed in CRC patients with LS (20). c-MYC proto-oncogene (MYC) and Adenomatous polyposis coli (APC) have been indicated as initial factors of CRC. MYC, a basic-helix-loop-helix transcription factor binding to MAX, is involved in the regulation of the cell cycle, cell survival, metabolism, and ribosome biogenesis (27). The mutation in APC is attributed to the activation of the Wnt/β-catenin cascade and accelerated CRC processes (28). Compared with LOCRC, EOCRC is classified as the low-MYC expression group and dysregulated MYC contributes to CRC carcinogenesis in patients with EOCRC (29). APC, a tumor-suppressor gene, was also found in patients with EOCRC (30). These pieces of evidence prove that there are indeed differences in onset processes between EOCRC and LOCRC.

### 2.2 Different clinical symptoms of early-onset colorectal cancer

EOCRC exhibits differences in symptoms, pathogenesis sites, and clinical pathological features, with symptoms such as rectal bleeding, abdominal pain, and anemia being more common (21). Abdominal pain and rectal bleeding, the two most common symptoms, were observed in 47% and 46% of EOCRC, according to the study aggregated from 2014-2018 (31). The overall incidence of CRC, especially distal colon, and rectum cancer, has decreased in individuals older than 50 years (10). Studies have reported the occurrence of EOCRC mostly in the left colon and rectum, however, LOCRC tended to occur mostly in the right colon (32, 33). This difference may be explained by different embryologic structures of the left- and right-side colon. The right side of the colon develops from the midgut, while the left develops from the hindgut. In addition, the left- and right sides of the colon are supplied with two different blood vessels (34). EOCRC has been observed to be a poorly differentiated and advanced stage compared with LOCRC (32). Most CRCs are adenocarcinomas and comprise three key subtypes, including classical adenocarcinoma (CA), mucinous adenocarcinoma (MA), and signet-ring cell carcinoma (SRCC) (35). EOCRC is likely an SRCC and MA subtype (36, 37). Compared with classical adenocarcinoma, SRCC, and MA are positively associated with a higher possibility of lymphatic invasion, perineural infiltration, and a second primary malignancy (SPM) (36, 37). Patients with SRCC were also younger and the tumor was likely to be poorly differentiated (37). In a few studies, young patients were likely to have SPM (38). Similarly, the rate of SPM was also higher in patients with EOCRC in the first 6-11 months compared to patients with LOCRC (39). Distant metastasis is more frequent in EOCRC rather than in LOCRC (40). Moreover, with the liver being the primary organ of metastasis, nearly 14% of diagnosed EOCRC exhibited liver metastasis (LM) (40). Most patients with EOCRC were found to have distant metastasis or regionally advanced cancer at the time of diagnosis (41). However, studies showed that specific metastasis features were not independent of stage. The frequency of lymphovascular invasion was found to be higher only in stage 2, while peri-neural invasion was higher in stage 3 in EOCRC (42). Compared to LOCRC, patients with EOCRC also had a higher relapse rate and a worse survival rate (43, 44).

## 3 Distinct role of obesity in early-onset colorectal cancer

**Figure 2:**
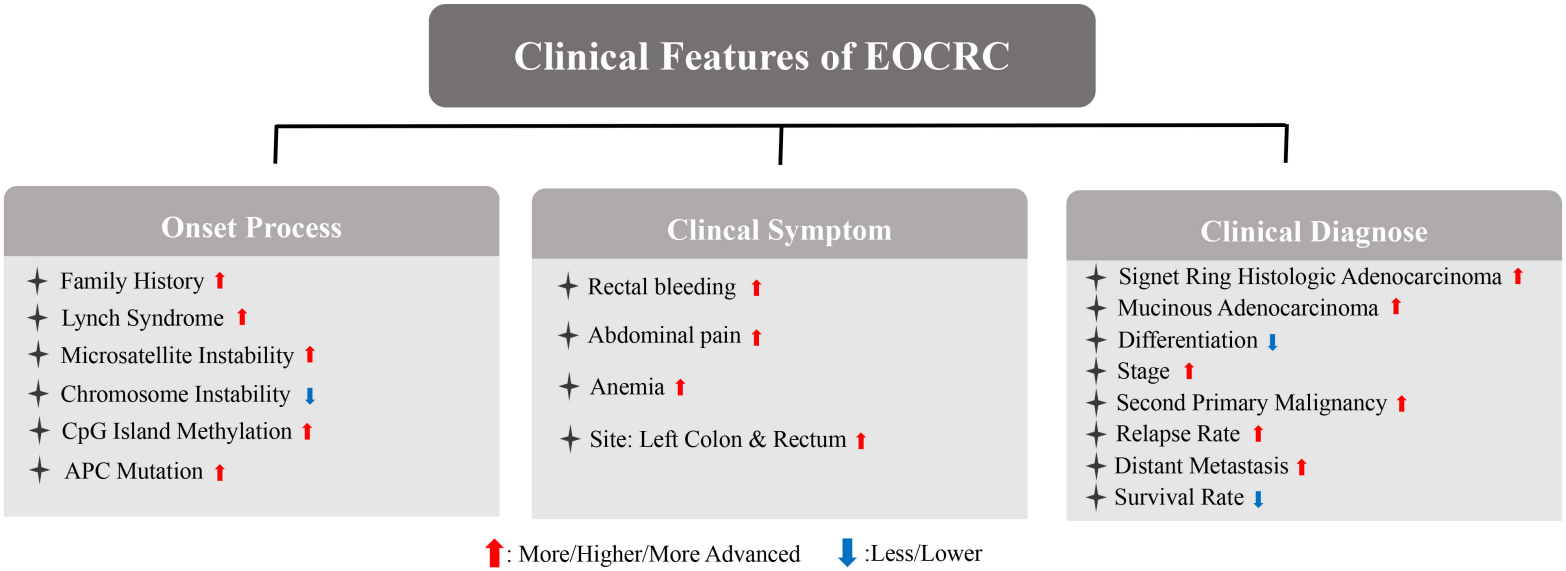
Association between obesity and EOCRC synopsizing the main clinical differences and biological mechanisms.

Even though an elevated incidence and a worse prognosis of EOCRC has been predicted in younger age, the prevention and early diagnosis of EOCRC still remain difficult as the causes of the disease are unknown (45). Multivariable analysis demonstrated that after controlling for staging and demographics, younger ages itself is no longer an indicator of worse outcomes (45). Therefore, the effect of age in EOCRC may be correlated with other risk factors. Interestingly, obesity, as a well-known CRC risk factor, correlated positively in younger patients with CRC (17). Nevertheless, the role of obesity in EOCRC and LOCRC still remains unclear. Obesity is highly correlated with metabolic syndrome, intestinal microbes, and inflammation (46, 47). In fact, low-grade inflammation also correlates with metabolic disorders and alterations of intestinal microbiota (48, 49). Therefore, we now summarize the clinical evidence and potential biological mechanism of obesity-induced EOCRC (**Figure 2**).

### 3.1 Clinical evidence between obesity and early-onset colorectal cancer

Patients with EOCRC are more likely to be obese. The degree of obesity is positively correlated with the occurrence and development of CRC, according to epidemiological evidence. Based on the Jass classification of CRC (MSI-H, CIMP-low/negative, BRAF-wildtype, and KRAS-wildtype), the large team of collaborating investigators found that only Jass type p5 or Lynch syndrome was not related to BMI (50). Therefore, obesity may play a potentially significant role in CRC. Regular physical activity and weight loss by bariatric surgery, respectively resulted in nearly 31% and 27% reduction in CRC risk, and obesity was identified as the risk factor for CRC (51, 52). Interestingly, reducing body weight at a young age decreased the risk of CRC in later ages (53). The average value of BMI in EOCRC patients, which is greater than 24, is significantly higher than that of patients with LOCRC (44). A retrospective study on 651 patients with EOCRC showed that 64.6% were overweight or obese (54). Weight gain in early- and mid-adulthood is associated with a significantly higher risk for CRC (55). Based on a propensity-weight analysis of 133008 patients with EOCRC, the association between EOCRC and a higher BMI (≥30kg/m^2^) was proved to be strong, with increased risks of advanced adenoma amongst obese EOCRC patients (36). Compared with sex, age, smoking, alcohol consumption, regular exercise, low-income status, and other risk factors of CRC, obesity is significantly associated with an increased risk of EOCRC. When compared with individuals with normal weight, the overweight group had an aHR of 1.10 (95% CI, 1.04–1.17), the obese group had an aHR of 1.19 (95% CI, 1.12–1.25), and the severely obese group had an aHR of 1.45 (95% CI, 1.31–1.61) for EOCRC (56). This evidence proved that patients with EOCRC are likely to be clinically obese and obesity may be a more dangerous factor in younger patients with CRCs compared with other risk factors of CRC. However, the potential mechanisms remain unclear.

Epidemiological evidence demonstrated that the incidence of EOCRC is triggered by environmental factors but not genetic evolutionary factors (17, 39, 57). Serrated polyps (SPs) and conventional adenomas (CAs) are important precursors of CRC. Interestingly, risks of SPs and CAs were significantly associated with obesity in young patients with CRC, while this association was weaker in aging CRC patients (58). Another study also showed that a higher BMI in early adulthood increased the incidence of serrated polyps and conventional adenomas (53). The high-fat diet, which is linked to obesity, is strongly associated with the specific molecular subtypes of EOCRC (59). Western diets and lack of activity, proven to accelerate the progression of obesity, also correlate with the rising incidence of EOCRC (60). High-sugar foods may accelerate *de novo* lipogenesis, obesity, and metabolic syndromes; accordingly, a prediction model showed the correlation of sweet and fried food with an increased risk of EOCRC (61). Moreover, in those EOCRCs patients having HNF1 mutation, high-fat diet (HFD) accelerates the formation of colonic polyps through the β-catenin/Cdx2 pathway (62). This specific subtype was also associated with conventional adenoma-carcinoma sequences, a well-known precursor of EOCRC (63). These studies emphasized the significant role of obesity in the initial process of EOCRC.

Obesity is also associated with postoperative complications. It distorts anatomical planes and reduces the operative space, resulting in technical difficulty and raising the anastomotic leak rate (64). Anastomotic leak was proven to increase local recurrence rates and decrease 5-year survival rates (64). Higher rates of shock, bleeding, wound disruption, postoperative infection, and digestive system complications are likely to appear in patients with obesity, which accelerates CRC progression by increasing the time of hospital stay and associated mortality (65).. Obesity is also positively associated with CRC recurrence and associated mortality rates (66, 67). Patients prediagnosed with an obesity in terms of BMI had worst outcomes than those with an overweight or normal BMI (68). Accumulation of body weight resulted in a higher proportion of visceral adipose tissue and subcutaneous adipose tissue, which are the principal deposition of fat (69). It was confirmed that visceral adipose tissue and subcutaneous adipose tissue increased the risk of CRC mortality (70). Interestingly, obesity also results in a rather worse outcome of EOCRC than of LOCRC. A systematic review and a multi-variable logistic regression model predicted BMI as an independent risk factor of EOCRC (71–73). A linear relationship of BMI with EOCRC was noted in a dose-response manner (71). One study of EOCRC in the UK showed that a higher BMI is correlated with a higher occurrence of tumor budding (TB) (74). TB is significantly associated with CRC metastasis, locoregional recurrences, and worse disease-free survival (75, 76). TB was also frequently found in SRCC, and this evidence corresponded with the specific histology of EOCRC (77). Patients with EOCRC have a higher perirectal fat area, which is strongly correlated with cancer metastasis and a worse survival (78). However, obesity results in a rather good outcome for LOCRC compared with EOCRC. In a recent study, a higher BMI was regarded as a protective factor of CRC prognosis, in which the participants were mainly patients with LOCRC (79). This inverse association proved that the risk of obesity in EOCRC was significantly increased compared with LOCRC. The association of obesity with EOCRC may be attributed to its involvement in the occurrence and development of CRC by affecting the metabolism and inflammatory factors, including insulin and insulin-like growth factors, sex hormones, and adipokines.

### 3.2 Obesity-associated metabolic disorder in early-onset colorectal cancer

A higher BMI is associated with an increase in metabolic disorders parallelly (80). Metabolic syndrome is a cluster of metabolic disorders, including obesity, diabetes mellitus type 2, hyperlipidemia, and hypertension, which showed a concurrent increase in incidence with the increase in obesity (80). Abnormal fat accumulation could also be seen in most individuals with metabolic syndrome, and excess fat plays a central role in such individuals (81). The pooled result of two research demonstrated that the cancer-specific mortality in CRC patients with metabolic syndrome was nearly two times higher than that of patients without metabolic syndrome (82). Metabolic syndrome may participate in the carcinogenesis of CRC through several mechanisms: i) insulin resistance, which leads to the accumulation of reactive oxygen species (ROS) and results in DNA damage and mutation; ii) increasing levels of leptin and adipokines promote the occurrence of CRC (83). Moreover, the CRC risk of metabolic syndrome in males was found to be higher than in females and this may result in estrogen and progesterone (83, 84). Studies have shown a greater sensitivity of EOCRC to obesity-induced metabolic syndrome compared to LOCRC. In one report, a history of metabolic disorders (including type 2 diabetes, mellitus, primary hypertension, disorders of lipoprotein metabolism and other dyslipidemias, overweight and obesity, or fatty change of liver) doubled the incidence of EOCRC (85). The number of metabolic disorders in patients was directly associated with the risk of EOCRC, increased risks of CRC are observed only among LOCRC patients who had more than three metabolic syndromes (86). Metformin, aspirin, statins, and other medications that treat metabolic syndrome can reduce the risk of CRC (87). The widespread use of these drugs for maintenance medication in obese patients with LOCRC might reduce the incidence of CRC compared with obese patients with EOCRC (87).

All metabolic syndrome components were shown to correlate independently with an increased risk of EOCRC (56). Obesity was present in almost 65%-80% of those diagnosed with type II diabetes (88). Increased body weight also impacts insulin clearance and results in insulin resistance, a kind of metabolic disorder (89). Dysglycemia resulted in a 20% to 38% increase in the risk of CRC (90). Weichuan Dong et al. suggested that diabetes has a stronger effect on EOCRC compared to LOCRC (91). The metabolic syndrome-related CRC might result in subsequent insulin resistance, which is linked to increased levels of insulin growth factor-1 (IGF-1) and IGFR (R) (92, 93). Elevated IGF-1 levels also induced CRC migration and proliferation via the phosphoinositide 3-kinase (PI3K)/Akt pathway (51, 94). Higher blood glucose increases the proliferation and metastases of CRC cells, and the insulin-growth factor receptor IGFR (R) pathway plays a pivotal role in the whole process (95). Dysglycemia is associated with the risk of EOCRC incidence and mortality. In one Swedish cohort study, patients diagnosed with diabetes had a 1.9-fold higher risk of developing EOCRC (39). Another research showed the correlation between sugar intake and a higher risk of EOCRC, while reduction of sugar intake was a protective strategy against EOCRC (96).

Hyperlipidemia, the subsyndrome of metabolic syndrome, showed no significant effects on CRC outcomes independently (82). However, one research showed that dyslipidemia was not associated with incident CRC (97). Another research showed the level of serum hyperlipidemia, was inversely correlated with the risk of colon cancer (98). However, one study including 1447 patients showed higher triglyceride levels in patients with EOCRC than those with LOCRC (99). Dyslipidemia was then found to have a strong association with EOCRC (OR=2.39, p<0.001) and was a risk factor (100). A meta-analysis of 20 studies demonstrated the correlation between hyperlipidemia (higher triglycerides and cholesterol levels) and obesity with EOCRC development (11). Likewise, in other studies, nearly 30% of patients with EOCRC were diagnosed with hyperlipidemia (80), and levels of HDL-c were also lower in EOCRC (99). These findings affirmed the important role of obesity-related hyperlipidemia in EOCRC.

Contrary to dysglycemia and hyperlipidemia, the role of hypertension in CRC is poorly understood (101). Nonetheless, one large prospective cohort study showed that hypertension is not associated with the risk and mortality of CRC (102). However, the Dietary Approaches to Stop Hypertension diet can reduce morbidity and prolong the survival of patients with CRC (103). Therefore, the exact role of hypertension in CRC still remains unclear. However, several cohort studies shown that hypertension was a risk factor in EOCRC compared with the normal cohort; surprisingly, hypertension also functioned as a protective role in EOCRC compared with LOCRC (73, 104). Moreover, EOCRC was likely to exhibit MSI, and hypertension was regarded as clinically independent of MSI status in CRC (105).

### 3.3 Obesity-induced dysfunction of intestinal microbiota in early-onset colorectal cancer

The intestinal microbes have crucial roles in intestinal homeostasis and are perceived as the second genome. The colonic microbial system comprises various microbes (bacteria, fungi, viruses, eukaryotes, and archaea) and play an important role in the development of CRC (106, 107). Intestinal microbes accelerate the occurrence of CRC via immune-regulation, gene interaction, inflammation, and other mechanisms (108). The abundance of intestinal microbes, including Fusobacterium nucleatum, enterotoxigenic Bacteroides fragilis, and Escherichia coli, increases significantly in the intestine (109). The interaction between microbiota and mitochondria also promotes the initial carcinogenesis of CRC. In one study, Peptostreptococcus anaerobiusactivate was found to activate toll-like receptor 2 (TLR2) and TLR4 to increase intracellular levels of ROS, contributing to cholesterol biosynthesis and cell proliferation in the development of CRC (110). Furthermore, two species of Propionibacterium were correlated with loss of mitochondrail transmembrane proteins and generation of ROS in CRC cells (111, 112). Tumor development and CRC metastasis are complex processes involving the tumor microenvironment (TME), of which, the microbes are the main component (113). Intestinal microbes invade CRC tissues and change the TME (114).

The intestinal microbiome regulates aging-related changes in cognitive function, innate immunity, and inflammation (115). The increasing incidence of EOCRC is multifactorial, while the microbiome is the key factor (116). In fact, the microbial diversity of patients with EOCRC was lower than those of patients with LOCRC (109, 117). Through metagenomic sequencing analysis, another study also showed significantly lower fecal alpha diversity in EOCRC compared with LOCRC and healthy individuals, indicating that intestinal microbes may be of great importance in EOCRC (117). The Flavonifractor plautii was enriched in EOCRC and exhibited a positive association with cancer metabolites (115). Therefore, the interaction between obesity and EOCRC may be based on intestinal microbes. Obesity can alter the intestinal microbiome, consequently leading to dysbiosis and metabolic dysfunction (118). Obesity-induced by HFDs leads to intestinal dysbiosis and the accumulation of toxic substances, such as lipopolysaccharides (LPS), secreted by intestinal microbes (119). *In vivo*, HFD resulted in the accumulation of LPS in EOCRC, which promoted CRC (60, 120). Cross-sectional research showed higher blood levels of LPS in patients with adenomas than in normal individuals. Moreover, levels of LPS in individuals with villous adenomas were higher than those with tubular adenomas (107). However, the link between dysbiosis in obese patients and its associations with EOCRC remains difficult to state, and require further investigations.

A sequencing of 16s ribosomal RNA gene revealed the differential components of intestinal microbes between patients with EOCRC and LOCRC (121). Firmicutes, Blautia, and Clostridia are three species abundant in EOCRC, and are positively correlated with HFD-induced obese individuals (81). The abundance of Firmicutes was higher in ob/ob mice (81). Clostridia plays a key role in fat accumulation and metabolic syndrome. Likewise, Blautia is positively correlated with blood levels of triglycerides, diglycerides, and Cardiolipin, which are common lipids in obese individuals (122). Clostridia also regulates the function of CD36, which is associated with lipid absorption (123). Actinomyces, identified as the key microbiota in the EOCRC group, was also abundant in obese individuals (109, 124). Bifidobacteriaceae was higher in older patients with CRC (121), and negatively associated with levels of total cholesterol, low-density lipoprotein-1 (LDL-1), and LDL-2. This evidence demonstrated the lesser significant role of obesity in LOCRC (125). Xiong. et al. indicated Fusobacteria and Bacteroidales as independent markers of EOCRC and LOCRC (117). Fusobacterium and Bacteroidales are both the pathobiont in individuals with CRC, but they perform differently in response to obesity (126, 127). The abundance of Fusobacterium increases significantly in obese people, and that of *Bacteroidales* decreased in glucocorticoid-induced obesity (128, 129). *Fusobacterium* also induce inflammation and suppresses host immunity in CRC, the abundance of *Fusobacterium* also correlates with shorter survival of patients with CRC (126). The levels of short-chain fatty acids (SCFAs) decrease in obese patients with CRC and have anti-cancer roles (130, 131). In fact, Mitsuokella. multacida, a bacterium that produced SCFA in the intestine, was absent in patients with EOCRC (60). This finding, based on intestinal SCFA levels, indicated that the risk of obesity may be higher in EOCRC. Thus, patients with EOCRC tend to exhibit obesity-related changes in gut microbes, suggesting a potential role of obesity in EOCRC.

### 3.4 Obesity-induced inflammation in early-onset colorectal cancer

Rather than attributing microbiome dysbiosis and metabolic syndrome, obesity was regarded as a cause of inflammation (60, 132). Therefore, a proposed mechanism linking obesity and cancer involves obesity-induced low-grade inflammation (133). Later, the state of low-grade chronic inflammation was regarded as a risk factor for CRC and causes DNA and protein damage in the occurrence and development of CRC (132, 134). Markers of systemic inflammation are also elevated in obese patients with CRC metastasis and correlate with a worse prognosis (68). Similarly, inflammation is a higher risk factor for CRC in youth rather than in middle-aged and older patients (132). Therefore, obesity-induced inflammation may have a more important role in EOCRC carcinogenesis (135). In one study, obesity-induced low-grade systemic inflammation in the carcinogenic milieu of CRC, was associated with the generation of inflammatory cytokines through the interaction between infiltrating immune cells and adipocytes (136). Another study showed that systemic chronic inflammation accelerates oncogenic processes in the colon through the tumor-associated macrophages, which is associated with tumor growth and worse survival in patients with EOCRC (45). Leptin and adiponectin, two well-known hormones related to obesity, are associated with macrophage metabolism and polarization towards a proinflammatory state (137). Therefore, the distinct effect of obesity in EOCRC might account for inflammation.

Apart from systemic inflammation, intestinal inflammation is also widely known as a CRC-promoting factor, especially in inflammatory bowel disease (IBD)-related CRC (138). IBD causes chronic nonspecific inflammation in the intestinal tract (139). Studies have shown a poorer differentiation of IBD-related CRC compared with sporadic CRC, however, the tumor stage and survival rate did not differ significantly (140, 141). However, when patients were categorized by age, those younger than 50 had worse survival in the IBD-related CRC group (138, 142). Patients with EOCRC were more likely to have IBD than healthy individuals (143). Accordingly, a cohort study from 2011 to 2017 showed that patients with EOCRC have a higher rate of IBD than patients with LOCRC and the prevalence of IBD in EOCRC was nearly seven times as much in LOCRC (60, 144). Patients with IBD also had a threefold risk of developing EOCRC rather than those with LOCRC (143), and a sixfold higher incidence of EOCRC (85). Vajravelu et al. ranked inflammatory bowel disease, rather than a family history of CRC/polyps, as the important risk factor for EOCRC (31). Besides the clinical evidence, Arif et al. demonstrated that IBD-related EOCRC has a higher rate of perineural metastasis, poorer differentiation, and lymphovascular invasion (140). Therefore, IBD was confirmed as a risk factor for EOCRC, although the incidence of EOCRC remained lower in Asia than in the West (56). Interestingly, one retrospective study showed ulcerative colitis, as a stronger hazard factor of EOCRC compared with Crohn’s disease (145). Apart from a traditional normal-adenoma-adenocarcinoma sequence of CRC, colitis-associated CRC (CAC) follows the inflammation-dysplasia-carcinoma sequence and inflammation is regarded as the core process in CAC carcinogenesis (146). CAC correlates with intestinal inflammation, and has been proven to promote genetic alteration, oxidative stress-induced DNA damage, and immune dysregulation, indicating a worse prognosis than patients with no history of IBD (147, 148). Even though IBD and metabolic disorders were proven to be risk factors in obesity-related EOCRC, patients with IBD and having metabolic disease exhibited a lower incidence of EOCRC compared with those without metabolic disease (85).

## 4 Conclusions and future directions

However, it is crucial to recognize that not all individuals with EOCRC engage in unhealthy lifestyles or present with risk factors commonly linked to the condition. This observation suggests the existence of alternative etiological factors that may contribute to the pathogenesis of EOCRC in such individuals. A genetic predisposition, characterized by germline mutations in high-penetrance cancer susceptibility genes, is likely to be more prevalent among individuals with EOCRC, irrespective of the absence of overt lifestyle risk factors (149). For instance, germline mutations affecting DNA repair genes, exemplified by those observed in Lynch syndrome, can precipitate a heightened susceptibility to CRC at a younger age, independent of lifestyle elements (150). Furthermore, the gut microbiome and its interactions with host factors are gaining recognition as significant contributors to the etiology of CRC, encompassing early-onset presentations (151).

In recent decades, there has been a persistent increase in both the incidence and mortality of EOCRC, and the features of EOCRC are distinct from LOCRC. Compared to LOCRC, the risk of obesity appears to be higher in EOCRC. This review provides an insight into the possible mechanism of obesity-EOCRC interactions. The crosstalk between EOCRC and obesity is supported by clinical evidence, gut microbe, metabolic syndrome, and inflammation. Interestingly, glucagon-like peptide-1 (GLP-1) agonists play an effective role in treating type 2 diabetes and preventing obesity (152). The application of GLP-1 agonists might be a potential therapy for EOCRC. Therefore, future studies may focus on obesity in the prevention and treatment of EOCRC.

## Author contributions

PX: Writing – original draft. ZT: Writing – original draft. HY: Writing – review & editing. CZ: Writing – review & editing.

## Glossary

**Table:**

AL	Anastomotic leak
APC	Adenomatous polyposis coli
BMI	Body mass index
CA	Classical adenocarcinoma
CAC	Colitis-associated CRC
CAs	Conventional adenomas
CD	Crohn’s Disease
CIMP	CpG island methylation
CIN	Chromosome instability
CL	Cardiolipin
CRC	Colorectal cancer
DASH	Dietary Approaches to Stop Hypertension
DGs	Diglycerides
DM2	Diabetes mellitus type 2
DPP4	Dipeptidyl peptidase 4
dMMR	Mismatch repair deficiency
EOCRC	Early-onset colorectal cancer
GLP-1	Glucagon-like peptide 1
GLP-1RAs	GLP-1 receptor agonists
GIP	Glucose-dependent insulinotropic polypeptide
HLD	Hyperlipidemia
HTN	Hypertension
IBD	Inflammatory bowel disease
IGF-1	Insulin growth factor-1
LM	Liver metastasis
LOCRC	Late-onset colorectal cancer
LPS	Lipopolysaccharides
LS	Lynch syndrome
LVI	Lymphovascular invasion
MA	Mucinous adenocarcinoma
MetS	Metabolic syndrome
MSI	Microsatellite instability
MYC	c-MYC proto-oncogene
PFA	Perirectal fat area
PI3K	Phosphoinositide 3-kinase
PN1	Peri-neural invasion
ROS	Reactive oxygen species
SAT	Subcutaneous adipose tissue
SPM	Second primary malignancy
SPs	Serrated polyps
SRCC	Signet-ring cell carcinoma
TB	Tumor budding
TGs	Triglycerides
TME	Tumor microenvironment
T2DM	Type 2 diabetes mellitus
UC	Ulcerative Colitis
VAT	Visceral adipose tissue
